# Draft Genome Sequences of *Acinetobacter baumannii* Isolates from Wounded Military Personnel

**DOI:** 10.1128/genomeA.00773-16

**Published:** 2016-08-25

**Authors:** Brock A. Arivett, Dave C. Ream, Steven E. Fiester, Destaalem Kidane, Luis A. Actis

**Affiliations:** a Department of Microbiology, Miami University, Oxford, Ohio, USA; b Biology Department, Middle Tennessee State University, Murfreesboro, Tennessee, USA

## Abstract

*Acinetobacter baumannii* is a Gram-negative bacterium capable of causing hospital-acquired infections that has been grouped with *Enterococcus faecium*, *Staphylococcus aureus*, *Klebsiella pneumoniae*, *Acinetobacter baumannii*, *Pseudomonas aeruginosa*, and *Enterobacter* species as ESKAPE pathogens because of their extensive drug resistance phenotypes and increasing risk to human health. Twenty-four multidrug-resistant *A. baumannii* strains isolated from wounded military personnel were sequenced and annotated.

## GENOME ANNOUNCEMENT

The Gram-negative coccobacillus *Acinetobacter baumannii* is an opportunistic human pathogen causing myriad human diseases, including pneumonia, bacteremia, urinary tract infections, meningitis, and wound infections. *A. baumannii* is the fifth most common Gram-negative pathogen associated with nosocomial infections (1, 2). Of concern is the increasing multidrug resistance of *A. baumannii* isolates, which has caused this bacterium to be included as an ESKAPE (*Enterococcus faecium, Staphylococcus aureus, Klebsiella pneumoniae, Acinetobacter baumannii, Pseudomonas aeruginosa*, and *Enterobacter* species) pathogen, underscoring its ability to “escape” antimicrobials (3). In fact, *A. baumannii* strains resistant to all known antibiotics have been encountered, demonstrating the paramount impact of this pathogen on public health (2). The genomes of 24 *A. baumannii* strains isolated from wounded warriors at Walter Reed Army Medical Center (WRAMC) and San Antonio Military Medical Center (SAMMC), Fort Sam Houston, San Antonio, TX, were sequenced using next-generation sequencing for future analyses to investigate the resistance and virulence mechanisms of this emerging pathogen.

As described previously, strains were routinely stored at -80°C in 10% glycerol (4). DNA was isolated from overnight LB cultures grown with agitation at 37°C using the DNeasy blood and tissue kit (Qiagen, Valencia, CA, USA). Absorption at 260 nm and 280 nm was measured for each sample to determine quantity and quality using the NanoDrop 2000 (Thermo Scientific, Wilmington, DE, USA). DNA concentrations for library preparation were determined by the SYBR green (Life Technologies, Grand Island, NY) standard curve method in a black 96-well plate (Corning, Tewksbury, MA, USA) using a FilterMax F5 spectrophotometer with multimode analysis software version 3.4.0.25 (Molecular Devices, Sunnyvale, CA, USA). Whole DNA was sheared to approximately 500 bp in a microTUBE-50 using an M220 focused ultrasonicator (Covaris, Woburn, MA, USA). Fragmentation of the resultant libraries was examined with a Bioanalyzer 2100 high-sensitivity DNA analysis kit (Agilent Technologies, Santa Clara, CA, USA) using version B.02.08.SI648 software. Individual libraries were normalized, pooled, and then sequenced using the MiSeq version 3 600-cycle kit (Illumina, San Diego, CA, USA) to perform 300-bp paired-end sequencing on a MiSeq instrument (Illumina), per the manufacturer’s instructions. *De novo* assembly was performed using Genomics Workbench 8.0 with the Bacterial Genome Finishing module (CLC bio, Boston, MA, USA) run on a workstation with an AMD Opteron 2.10 GHz 16-core processor with 128 Gb DDR3 ECC random access memory (RAM). Genomes were annotated with Prokka version 1.10 on a quad-core i7 workstation with 32 Gb DDR3 running Ubuntu 14.04 LTS (5). The *de novo* assembly statistics for 24 *A. baumannii* isolates are shown in Table 1.

**TABLE 1:** Assembly metrics and accession numbers of *A. baumannii* genomes

Strain ID	No. of contigs	*N*_50_ contig size (bp)	Total size (bp)	Coverage (×)	G+C content (%)	No. of ORFs^a^	No. of RNAs	Accession no.
AB2828	107	124,070	4,426,896	30	39.21	4,274	53	LRDT00000000
AB3340	76	132,604	4,010,248	28	38.86	3,864	49	LRDU00000000
AB3560	58	247,914	4,012,126	30	38.92	3,894	59	LRDV00000000
AB967	27	401,652	3,795,032	29	38.84	3,633	62	LRDS00000000
AB3785	70	134,647	3,894,584	29	39.01	3,745	58	LRDX00000000
AB3638	78	108,414	4,294,582	31	38.72	4,113	62	LRDW00000000
AB3806	86	96,852	4,295,294	33	38.75	4,117	59	LRDY00000000
AB3927	45	227,995	4,113,781	30	38.82	3,978	58	LRDZ00000000
AB4026	67	160,728	3,905,198	30	38.99	3,749	50	LREB00000000
AB4027	72	152,887	3,903,961	32	39.00	3,749	54	LREC00000000
AB4025	69	152,887	3,902,672	29	39.00	3,741	55	LREA00000000
AB4456	58	182,799	4,001,807	27	38.92	3,857	47	LREF00000000
AB4052	43	262,160	3,921,338	33	39.00	3,739	51	LRED00000000
AB4448	43	369,360	3,992,257	28	38.92	3,854	58	LREE00000000
AB4490	98	84,980	3,947,403	31	38.99	3,786	60	LREG00000000
AB4498	76	128,212	3,905,177	32	39.00	3,753	57	LREH00000000
AB4795	78	113,293	3,882,341	33	39.03	3,727	62	LREI00000000
AB4878	45	223,470	3,862,567	26	38.98	3,685	50	LREJ00000000
AB4957	50	223,470	3,882,040	33	38.97	3,722	60	LREL00000000
AB4932	39	237,199	3,865,974	33	38.99	3,703	60	LREK00000000
AB5001	33	223,470	3,789,469	30	38.99	3,586	52	LREN00000000
AB4991	52	310,788	3,877,107	28	39.09	3,686	58	LREM00000000
AB5674	34	419,504	3,869,253	29	39.03	3,679	52	LREP00000000
AB5197	58	184,472	3,959,484	33	39.04	3,799	58	LREO00000000

a ORFs, open reading frames.

### Accession number(s).

The whole-genome shotgun projects were deposited into GenBank under BioProject ID PRJNA261239 with accession numbers listed in Table 1.
